# Surviving the Struggle of COVID-19: Practical Recommendations for Pediatric/Adult Cardiology and Cardiac Surgical Programs in Resource-Limited Settings: a Review

**DOI:** 10.21470/1678-9741-2021-0477

**Published:** 2022

**Authors:** Kevin Pilarczyk, Vinicius Nina, Lynn Boshkov, Barbara Ferdman, Emily A Farkas, Nicole Burnham, Renzo Cifuentes, Daniel Ntogwiachu, Aubyn Marath

**Affiliations:** 1 RobinAid Foundation, Hamburg, Germany.; 2 Department for Intensive Care, imland Klinik Rendsburg, Rendsburg, Germany.; 3 Department for Cardiothoracic Surgery, Universidade Federal do Maranhão, São Luís, Maranhão, Brazil.; 4 Cardiostart International, Tampa, United States of America.; 5 Division of Hematology, Department of Medicine, Oregon Health & Science University, Portland, Oregon, United States of America.; 6 Pediatric Cardiology, CardioStart International, Tampa, Florida, United States of America.; 7 School of Medicine, Indiana University, Indianapolis, Indiana, United States of America.; 8 Children’s Hospital Canada, Vancouver, Canada.; 9 Division of Thoracic Transplantation and Mechanical Circulatory Support, Department of Surgery, Miller School of Medicine, University of Miami, Miami, Florida, United States of America.; 10 African Federation of Critical Care Nurses.

**Keywords:** Covid-19, SARS-CoV-2, Pandemic, Cardiac Services, Health Strategies, Information, Medical Societies

## Abstract

**Introduction:**

The primary aim of this systematic review is to provide perioperative strategies to help restore or preserve cardiovascular services under threat from financial and personnel constraints imposed by the coronavirus disease 2019 (COVID-19) pandemic.

**Methods:**

The Medical Literature Analysis and Retrieval System Online, Excerpta Medica dataBASE, Cochrane Central Register of Controlled Trials/CCTR, and Google Scholar were systematically searched using the search terms “(cardiac OR cardiology OR cardiothoracic OR surgery) AND (COVID-19 or coronavirus OR SARS-CoV-2 OR 2019-nCoV OR 2019 novel coronavirus OR pandemic)”. Additionally, the webpages of relevant medical societies, including the World Federation Society of Anesthesiologists, the Cardiothoracic Surgery Network, and the Society of Thoracic Surgeons, were screened for relevant information.

**Results:**

Whereas cardiac surgery and cardiology practices were reduced by 50-75% during the pandemic, mortality of patients with COVID-19 increased significantly. Healthcare workers are among those at high risk of infection with COVID-19.

**Conclusion:**

Hospitals must provide maximum protective equipment and training on how to use it to healthcare workers for their mutual protection. Triage management of patients — which accounts for patient’s clinical status and risk-factor profile relatable to which services are available during the COVID-19 pandemic — is recommended. A strict reorganization of the hospital resources including preoperative, intraoperative, and postoperative detailed protective measures is necessary to reduce probability of vector contamination, to protect patients and the cardiovascular teams, and to permit safe resumption of cardiological and cardiac surgical activity.

**Table:** 

Abbreviations, Acronyms & Symbols	
ACS	= Acute coronary syndrome
AGPs	= Aerosol-generating procedures
AHA	= American Heart Association
CADR	= Clean air delivery rate
CDC	= Centers for Disease Control and Prevention
COVID-19	= Coronavirus disease 2019
CVD	= Cardiovascular disease
EAPCI	= European Association of Percutaneous Cardiovascular Interventions
ECG	= Electrocardiogram
ESC	= European Society of Cardiology
HEPA	= High-efficiency particulate air
ICU	= Intensive care unit
IgG	= Immunoglobulin G
IgM	= Immunoglobulin M
LMICs	= Low- and middle-income countries
MeNTS	= Medically necessary, time-sensitive
MRI	= Magnetic resonance imaging
NGOs	= Non-governmental organizations
NSOAPs	= National Surgical, Obstetric, and Anesthesia Plan
OR	= Operating room
PCR	= Polymerase chain reaction
PPE	= Personal protective equipment
RHD	= Rheumatic heart disease
RT	= Respiratory therapist
SARS-CoV-2	= Severe acute respiratory syndrome coronavirus 2

## INTRODUCTION

The coronavirus disease 2019 (COVID-19) pandemic has made a global imprint that has left almost no one untouched. As of August 2021, over 215 million people are confirmed to have been infected with COVID-19, with more than 4.6 million deaths, resulting in national and regional lockdowns and other emergency contingency measures across the globe. It is probable that this understates the true level in view of relative isolation in many communities. In tertiary care centers around the world, since March 2020, many elective surgical procedures have been postponed or cancelled in large numbers, which leads to a backlog of nearly 30 million procedures in just 12 weeks. In low- and middle-income countries (LMICs), where surgical care delivery is already constrained by reduced availability of workforce, infrastructural capacity, geographical distance, and financial barriers, the COVID-19 pandemic may have even larger consequences.

Cardiac teams attempting to perform surgeries are now facing an unprecedented challenge from COVID-19 infection’s effects on the cardiovascular system. Among the wide variety of threats to health, cardiovascular disease (CVD) is now the leading cause of death in the world and is responsible for nearly one-third of all global deaths. Since the year 2000, an increase in an individual’s lifespan has become evident, but one-third of CVD deaths have occurred in those aged between 30 and 70 years — the most economically productive section of a country’s community. In some locations, age-standardized CVD mortality rates showed a nearly six-fold higher level in 2019. In the African continent, CVD has become the leading cause of death for the first time in the history of the global disease estimates.

Six billion people in LMICs lack timely or ready access to safe and affordable cardiac surgical care when needed; it has a low priority on the global public health and global surgery agenda^[1]^. Some major hospitals are now threatened with closure. The immense shortage of even rudimentary equipment for viral and general testing and clinical management, even at baseline levels, that was applied prior to the pandemic now severely limit the ability of cardiovascular teams to try to follow “gold standard” practices. Diversion of precious resources and funds from their programs is already taking place. For many, livelihoods have been upended and elective procedures suspended indefinitely; some are experiencing a reduction in pay to keep support staff and practices afloat while others are now receiving no revenue at all.

## METHODS

### Search Strategy and Selection of Sources of Evidence

We conducted a systematic search of the Medical Literature Analysis and Retrieval System Online (or MEDLINE), Excerpta Medica dataBASE (EMBASE), Cochrane Central Register of Controlled Trials (or CENTRAL/CCTR), and Google Scholar using the combination of medical subject headings (or MeSH) and keywords “(cardiac OR cardiology OR cardiothoracic OR surgery) AND (COVID-19 or coronavirus OR SARS-CoV-2 OR 2019-nCoV OR 2019 novel coronavirus OR pandemic)”. Additionally, the webpages of the following medical societies were screened for relevant information: The World Federation Society of Anesthesiologists, the Cardiothoracic Surgery Network, the Society of Thoracic Surgeons COVID-19, Centers for Disease Control and Prevention (CDC), American Society of Anesthesiologists, Society of Critical Care Medicine, European Society of Cardiology (ESC), American College of Cardiology, and American Heart Association (AHA).

### Eligibility Criteria

Only articles written in English that reported relevant aspects of perioperative management of patients with COVID-19 were included. Two reviewers (KP and AM) conducted the search independently and screened all article types for eligibility using their titles and abstracts. Duplicate and irrelevant articles were excluded. Articles that did not address the primary objective and those that were correspondences and editorials were also excluded.

## RESULTS

### Current Evidence

COVID-19 is particularly important for cardiac and intensive care teams in LMICs:

- Clinical infection with COVID-19 is associated with a higher morbidity and mortality in patients undergoing surgery^[2]^.
- COVID-19 may affect and impact on the previously healthy cardiovascular system and vital organs^[3]^ (Figure 1).
  **Fig. 1:**
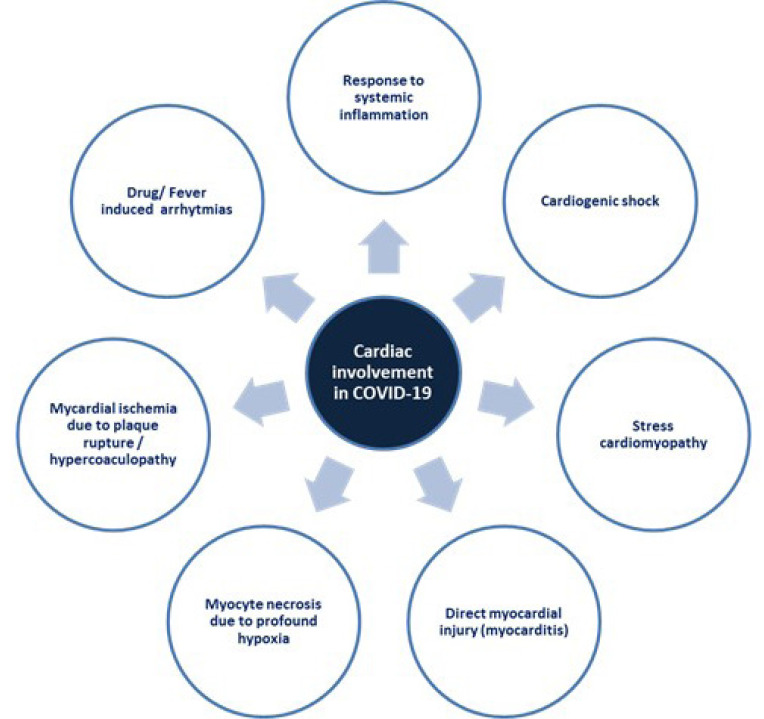
Cardiac involvement in coronavirus disease 2019 (COVID-19).
- COVID-19 may cause symptoms that mimic symptoms seen in other pulmonary disease or CVD presentations^[3]^.
- Cardiac care team members are at risk for acquiring COVID-19, and it may play a role in spreading the disease between patients and within their communities^[4]^.
- Patients with congenital heart defects are known to have higher risk for complications with viral illness^[5]^.
- COVID-19 is having a profoundly negative impact on the care of patients with CVD^[5]^.
- Triage and prioritization of patients scheduled for cardiac interventions have already become necessary^[6,7]^.

### Accomplishing Cardiac Surgery During the COVID-19 Pandemic

Widely different situations are being faced now, especially in LMICs. Effects of the COVID-Pandemic on cardiac care in LMICs are summarized in Table 1.

**Table 1:** Assessment of access to cardiac surgical care in low- and middle-income countries (LMICs) before and during the coronavirus disease 2019 (COVID-19) pandemic, and opportunities for post-COVID-19 cardiac surgical scaling.

	Pre-COVID-19	During COVID-19	Post-COVID-19?
**Accessibility**	~4,000 cardiac centers	Lockdowns and travel restrictions impede travel to regional centers	Temporary regionalization of cardiac care
	Few, if any, centers in LMICs	No “hot” *vs.* “cold” centers	Inclusion of cardiac care within NSOAPs
	Regionalization of centers within and between LMICs		
**Capacity**	High-income countries have 180× higher cardiac surgeon density than low-income countries	Shift to COVID-19 care	Government collaborations to train cardiac surgeons and cardiac teams in countries with existing training programs
	NGO and visiting team support	Use of resources for COVID-19 care	Optimizing supply chains in collaboration with industry
		Risk of health worker infection	Fostering local economies of scale
**Quality**	Widely variable outcomes mostly defined by resource constraints	COVID-19 complicates postoperative disease course	Maintain and expand quality improvement mechanisms to support pre, peri, and postoperative care delivery
	Excellent outcomes suggest feasibility of cardiac surgery	Limited knowledge of COVID-19 in cardiac surgery	
**Affordability**	Limited financial risk protection	Furloughs, unemployment, limited government support	Integration of cardiac surgical care in universal health coverage and financial risk protection models
	High procedural costs relative to living standards	Reduced subsidized services by NGOs and visiting teams	Innovative financing mechanisms
	Subsidized procedures by NGOs and visiting teams		

NGOs=non-governmental organizations; NSOAPs=National Surgical, Obstetric, and Anesthesia Plans

Four major determinants that directly influence receiving surgical care are:

- Accessibility to tertiary care.
- Affordability by the hospital and patients who seek help.
- A program’s current capacity.
- Ensuring quality of service.

### Accessibility

As a result of lockdowns and travel restrictions, cardiac centers have been less able to treat patients from other countries with little or no access to cardiac surgery.

Some LMICs rely on only one or two centers for the entire country for tertiary care; these must now cope with a surge in COVID-19 infections nearby. Fear of being stigmatized or marginalized result in people seeking medical care late or in a critical condition. Misinformation and rumors about COVID-19 and vaccination are also keeping people away from hospitals when they are ill.

### Affordability

Lockdowns, furloughs, dismissals, and reallocation of essential staff are resulting in reduced services in many sectors; millions of people worldwide have lost their jobs. In some cases, these measures have taken away the single source of income of individuals and their entire families. Government cutbacks in budget allocation per capita in LMICs have resulted in very limited financial support for tertiary care hospitals at this time of crisis. The lower surgical volume centers may inevitably suffer increased procedural costs due to lower turnover and increasing overhead costs. The reduction in visits by international visiting teams has reduced the number of philanthropic (subsidized) cases. Reports of patients dying on the waiting list are increasing; families are facing a worsening financial situation. Where patients have to pay for part of their in-hospital care (*e.g.*, medications), surgery may become impossible to consider. The CVD burden is worsening and is continuing its decades-long rise for almost all low-income countries, doubling from 271 million to 523 million^[8]^.

### Capacity

The scarcity of health workers in LMICs has led to a shift of specialists to care for critically ill patients. Conversion of operating rooms (OR) and intensive care unit (ICU) beds has been carried out to handle rapid growth in complicated COVID-19 cases. Previously contracted supply sources and surgical donations have been curtailed by border closures and travel restrictions, reducing the availability of equipment and consumables. An elevated infection risk for health workers, especially those involved with invasive care, has become significant with COVID-19 and may shift health workers from disease curers to vectors.

### Quality of Service

In some instances, the loss of the lead cardiac surgeon and other essential team members in LMICs has forced closure. The unique pathophysiological impact of COVID-19 has led to worse outcomes among COVID-19-positive patients, both for those infected before and after their operations. Reports are highlighting respiratory and coagulopathic complication rates after cardiac surgery as a result of COVID-19 infection, and some of these have produced a fatal outcome. There is still limited knowledge of the exact outcomes for patients undergoing different cardiac surgical procedures and how specific risk factors may affect outcomes.

## DISCUSSION

### Recommendations to Adopt

#### Adjusted Management of Cardiac Surgery in LMICs

Although our understanding of many features of COVID-19 are changing weekly, several medical societies have made recommendations for those in advanced centers^[9,10]^.Many of these advisory statements are applicable in LIMCs.

#### Triage of Cardiac Surgical Patients with Known/Suspected COVID-19

Several authoritative bodies have offered guidelines and recommendations on how to conserve resources and triage patients who need more urgent care^[6]^.^7]^. Clinical management of all chronic cardiac conditions is changing substantially. ESC has issued a guidance document on how to prioritize management in patients with cardiac conditions^[10]^. Patients with acute coronary syndrome (ACS), left main percutaneous coronary intervention, pacing battery replacement, and valvular heart diseases that are hemodynamically unstable should be considered emergent and should not be postponed.

#### Reduce Caseload

As hospitals become increasingly populated with either suspected or confirmed COVID-19 patients, separation of cardiac patients is needed with no staff crossover, to minimize risk of nosocomial infection. As a short-term measure, reducing the number of cardiac surgical procedures will help those needing ICU care for other reasons: vital equipment resources, ICU beds, ventilators, pharmaceuticals, personal protective equipment (PPE), and repurposing staff with advanced skills will be more available. A negative consequence is the risk of losing essential staff. Cardiac surgery requires a dedicated team of uniquely skilled individuals (cardiac OR scrub and circulators, perfusionists, cardiac anesthesiologists, and perioperative caregivers). Utilizing these individuals for non-essential operations or placing them elsewhere may increase their chances of COVID-19 exposure, and it will reduce their availability for future more urgent cardiac procedures that do arise.

#### Managing the New Caseload Reduction

A cardiac surgery acuity scale builds upon the widely accepted ACS Elective Surgery Acuity Scale by accounting for inpatients that require urgent or emergent treatment. It is worth implementing^[6,7,11]^. In addition to Tier 1 to 3 for elective interventions, the cardiac surgery acuity scale includes:

- Tier 4a: urgent surgery required to permit safe hospital discharge.
- Tier 4b: urgent surgery required within 24 to 48 hours to prevent clinical deterioration.
- Tier 5: emergent surgery required to prevent immediate death.

#### Use of a Medically Necessary, Time-Sensitive (MeNTS) Score

A scoring system for MeNTS procedures can facilitate decision making and triage in the setting of COVID-19, as recommended by Prachand et al.^[12]^.

They describe a scoring system that integrates factors, such as resource limitations and COVID-19 transmission risk, to providers and patients to guide triage for MeNTS procedures and weigh individual patient risks.

#### Evaluating Any Cardiovascular Effects of COVID-19

While severe acute respiratory syndrome coronavirus 2 (SARS-CoV-2) most commonly affects the respiratory system, some patients experience cardiovascular effects *with and without* symptoms^[13]^. Both effects may be evident in some patients who have pre-existing cardiac disease^[14]^. If available, myocarditis can be identified by cardiac magnetic resonance imaging (MRI)^[15]^. Elevated troponin levels or electrocardiogram (ECG) abnormalities were found in 7% to 28% of hospitalized patients with COVID-19 and were associated with poor outcomes^[14]^.

#### Managing Patients with COVID-19 and Elevated Troponin Levels

The European Association of Percutaneous Cardiovascular Interventions (EAPCI) position is recommended here — they issued a position statement on invasive management in patients with ACS during the COVID-19 pandemic^[16]^.

The EAPCI recommends that in cases of mild troponin elevation (< 2-3 times the upper limit of normal), particularly in older patients with pre-existing cardiac conditions, a work-up for type-1 myocardial infarction is not indicated, unless strongly indicated by clinical presentation and ECG findings.

#### Managing Myocardial Injury in COVID-19 Patients

Current guidelines proposed by Caforio et al.^[15]^ for the treatment of viral myocarditis are applicable in most settings, they include the use of standard heart failure therapies and supportive measures. An position statement of the European Society of Cardiology Working Group on Myocardial and Pericardial Diseases remains helpful in clinical management^[15]^.

Selective use of prednisolone has shown benefit in some case reports, however, there is insufficient evidence to support the *routine* use of such steroids in these patients, which may also cause harm.

COVID-19 infection has now clear association with abnormalities in blood clotting. Anticoagulation treatments to stop clotting could be beneficial though evidence to date is not based on wide analysis nor beyond observational studies.

We recommend follow-up review in patients who exhibit structural and functional abnormalities, they should have an echocardiogram 1-3 months after discharge and then monitored for a minimum of six months, with heart failure therapy tailored to damage observed and recovery delays. Monitored exercise testing may also be required and determined according to future employment type.

#### Other Forms of Heart Disease Adversely Affecting Outcome in LMICs

Rheumatic heart disease (RHD) regularly affects the poorest countries and their communities, posing potential complications from COVID-19 because of their links with cardiac health and functioning. Around 33.4 million people worldwide have been estimated to be suffering from rheumatic fever and ensuing RHD. At present, data is not fully available about the vulnerability of patients with RHD and the impact of COVID-19 infection. RHD patients may have left chamber dysfunction of the heart or elevated pulmonary pressure which may predispose them to complications of COVID-19.

#### Managing Chagas Disease During the COVID-19 Pandemic

Chagas disease, caused by a tropical parasite, affects around 6 million people. It may affect the heart in some patients. Those with Chagas disease should follow the same recommendations as the general population^[17]^.

#### Preoperative Surgical Screening and Work-up

Socioeconomically deprived patients require clinicians to be especially sensitive to the issues they face. Low socioeconomic status stands alone as an important risk factor for total mortality independent of any other risk factors. Lockdown, loss of job, spousal tensions and/or abuse, threat of dying on waiting list, threat of hospital closure, poor access to food, unavailability of essential medications, evolving mental health issues, fear of disease that may be spread within a hospital, and fears surrounding their perception of operative outcome are common. Viral infections are now also viewed as independent risk factors for cardiovascular events such as heart attack.

An essential part of clinical history-taking and examination by the cardiologist is the harmonious and disarming relationship that should be built within the first five minutes to reduce the reasonable anxiety of the patient.

Show one’s face on first greeting the patient, using appropriate distance or screen protection, should help. Hiding behind the mask and denying any physical contact obstructs that vital interaction — especially in children or young adults. During interview in the clinic, a workable compromise is to wear an N-95 mask and clear face-shield, then briefly pull the mask down to show one’s face before continuing the interview (and on completing it).

To perform perioperative echocardiography with pre- and post-comparisons (extending to three, and six months when indicated) may help demonstrate diffuse myocardial dyskinesia and pericarditis in COVID-19 suspected cases.

Complete COVID-19 testing close to the planned operative date (preferably < 48 hours) helps to lessen the risk that a patient becomes positive while waiting for a surgical procedure.

It is recommended to avoid emergency surgical procedures during off-duty hours, when possible, due to limited team staffing and the potential lack of optimal specialty-specific expertise.

On the day of interview, the patient’s pulmonary function should be assessed before surgery to assist with decisions about planning later separation from the ventilator postoperatively. Over 12% of COVID-19 patients may not develop a fever at first evaluation, nor at admission, nor be symptomatic until several days postoperatively^[18]^. Patients should be advised that undergoing surgery with perioperative or recent SARS-CoV-2 infection appears to be at increased risk of postoperative venous thromboembolism compared with patients with no history of SARS-CoV-2 infection.

If possible, a family relative should be selected by patients to be the *only* family member to accompany them to the hospital and be available throughout the perioperative period.

For both pediatric and adult patients, timing of and reducing the number of clinic visits helps avoid unnecessary exposure to other patients and the associated stress factors that may be present. A single evaluation performed by the cardiologist and respiratory therapist (RT) preoperatively at 12-14 days before surgery can help support a pre-admission assessment that yields most information. This should include standard COVID-19 testing, preoperative blood panel, temperature, chest X-ray, and respiratory and echo-cardiological assessments. Pre- and post-comparisons following operation may help demonstrate diffuse myocardial dyskinesia and pericarditis in COVID-19 suspected cases. The role of the RT is vital — in one report, patients with COVID-19 infection on ventilators developed more barotrauma than patients who required intubation for other reasons.

It is vital to ensure that essential PPE items are well stocked before surgical planning takes place.

The institution must have consistent *locally constructed* policies for staff, patients, and their relatives to follow and develop locally relevant teaching protocols for perioperative COVID-19 preventative measures, including nosocomial causes.

**Fig. 2:**
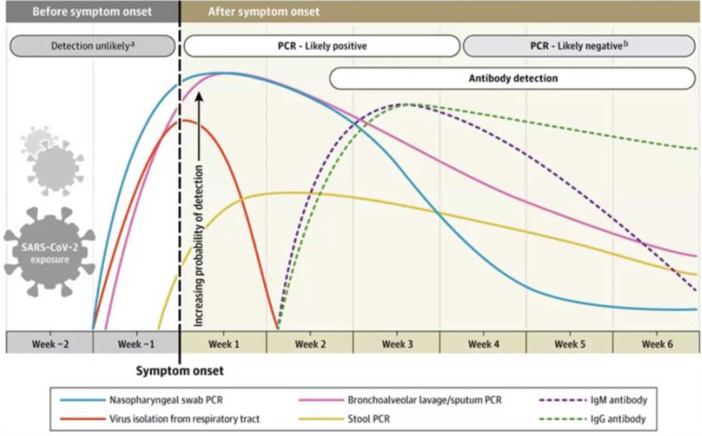
Interpreting diagnostic tests for coronavirus disease 2019 (COVID-19) (with permission, JAMA)^[19]^. IgG=immunoglobulin G; IgM=immunoglobulin M; PCR=polymerase chain reaction; SARS-CoV-2=severe acute respiratory syndrome coronavirus 2.

Decisions about the timing of performing elective surgery carry with them the uncertain knowledge that a patient has not already been exposed with COVID-19 in the interim period between evaluation and surgical admission. Family members may unwittingly be the cause. Sethuraman et al.^[19]^ (Figure 2) have illustrated the difficulty in the best timing of testing and capturing COVID-19 patients or excluding those who may be asymptomatic or symptomatic from non-COVID-19 causes.

In performing preoperative evaluations, there is shared symptomatology among COVID-19 disease presentations and other diseases. Those with CVD may be of older age, have diabetes, obesity, and hypertension and other diseases which are endemic to the region. Diseases caused by vectors may cause similar symptoms and signs and may also produce hemorrhagic changes making differential diagnosis more difficult. The now familiar COVID-19 cardiovascular symptoms of chest pain or palpitations, brain fogginess, headaches, and postural orthostatic tachycardia syndrome may also be present in those with cyanotic congenital heart disease, post-rheumatic valve, or ischemic heart disease. A clue to supporting a COVID-19 diagnosis is anosmia/dysgeusia (loss of smell or taste) which may be accompanied by other symptoms (cough, fever, dyspnea, musculoskeletal symptoms of myalgia, joint pain, fatigue, and varied gastrointestinal symptoms).

Enquiring about perceptions of measured distance walking in older adults may also help give information on oxygen saturation changes (with COVID-19 infected patients, this may drop precipitously with exertion).

On admission, the RT should manage the preoperative and postoperative care during the ICU stay. This strategy may not capture all those already infected with COVID-19, those who were exposed just prior to admission, or those who may later infect others, but it may reduce the number of people exposed.

If no test kits are available, it is safest to assume that the patient is infected and, if at all possible, continue separating the patient from other perioperative patients. If tests kits *are* available and the patients test positive for COVID-19, they are best moved to an area separate from other perioperative patients.

Nurses caring for patients diagnosed with COVID-19 or suspected to be infected should not take care of non-infected patients.

In choosing who to operate on after first ensuring availability of a large stock of essential disposables and determining urgency of the procedure, we recommend prioritization as suggested by Stephens et al.^[20]^.

In prioritizing patients, consideration to comorbidities and likely time requiring ventilatory support is important.

Telemedicine techniques should be used where possible. The development of telemedicine adjuncts via Zoom, Facetime, Skype, WebEx, Doximity, and other similar platforms are a welcome technological advance that make it possible for doctors and healthcare personnel to be more accessible to patients and their questions, and yet remain safe. For resource-poor locations struggling to develop their programs, however, internet access may not be easily available or reliable and may not have the bandwidth to support these platforms. Many patients in poor and remote locations may not have access to a computer or cellphone (except flip-up phones, that are sometimes provided by their employers).

COVID-19 preventative measures are sometimes difficult to teach remotely; the availability of hand-sanitizer may be limited, and hygienic bathroom facilities may be lacking. In resource-poor hospitals, lavatories may lack daily cleaning routines, antiseptic soap, and paper; their design features may encourage fomite transmission. The situation is usually worse in towns, bus stations, and other typically crowded areas so contamination levels can easily rise. People vary in their perception of how a virus can afflict an individual. The precautions of wearing masks and frequent hand washing may not be strictly observed nor presented as a required routine, and facilities are, in any case, less available there. International recommendations developed by the World Health Organization and followed in many individual centers of excellence should be implemented when possible to prevent an increase in infection among patients and colleagues^[21]^.

The priority is for doctors and other healthcare leaders to model their practice by example, and to use media and press releases to promote them.

#### Addressing Issues Inside the Hospital Operating Room

- Modern designs for laminar flow delivery which include regularly inspected and changed filters may not be available in LIMCs. In subtropical and tropical regions, a fan perched in the corner of the OR or ICU is sometimes used in an effort to decrease the room temperature to make working conditions more tolerable. It will, however, disperse contaminated droplets throughout a room.
- If possible, modifying the OR suite to allow foot or elbow-operated sliding doors guarding entry into the OR may reduce the fan effect of ordinary door opening. Currents of air can increase if outside corridor windows and doors are left open. Air purifiers or cleaners with a high-efficiency particulate air (HEPA) filter are necessary to remove viruses: they are more than 99.7% efficient. In a study of a hospital room occupied by two COVID-19 patients at the University of Florida Health, HEPA filters were attached to air sampling devices. Although SARS-CoV-2 ribonucleic acid was found in many of the patients’ exhaled air samples, it was absent in emergent air that had passed through the HEPA filters — suggesting that these devices do block the pathogen effectively^[22]^. Purifiers that are marketed locally should exhibit a clean air delivery rate (CADR) to which some countries require certification. It defines how quickly a purifier can rid the air of specific types of particles, including dust and smoke — which may be roughly in the same size range as aerosols bearing SARS-CoV-2. A HEPA purifier with a CADR score of 300 would clear 99% of particles from a room measuring 10 by 10 by 10 feet.
- Relevance: The vapor of the exhaled breath, sneezes, and coughs can project an aerosol missile of droplets up to 10 feet; existing air circulation, room temperature, and humidity level can extend that distance even further. The locally moist and warm atmosphere within the gas cloud allows virus-containing droplets to evade evaporation for longer than with isolated droplets. Under these conditions, the lifetime of a droplet can extend by a factor of up to 1000, from a fraction of a second to minutes^[22]^. They may stay suspended in the air for hours or fall onto equipment, depending on the airflow patterns or type of ventilation available. In laboratory-created respirable-sized aerosols, Fears et al.^[23]^ reported that the virus could retain infectivity for up to 16 hours. The impact of COVID-19 transmission is teaching us that our tendency to wander in and out of the OR and into the ICU — or outpatient area (and sometimes, into our car to go to the supermarket and home in our scrubs) — is a dangerous habit that should not continue^[24]^.
- Each team member should check if the mask they chose to use is adequate, whether or not they should come into work if symptomatic *from any cause*, and whether or not PPEs are available to them on arrival.
- Gowning up: The close physical space within which the surgical team operate on the heart patient may require a change in practice to follow the example of gowns and hoods worn by orthopedic teams during joint replacement. The exception to that is the requirement of loupe magnification glasses and headlights which cannot be easily used within a plastic visor.
- Fresh gowning upon entering the ICU is important and changing out of scrubs upon leaving the ICU or the OR may become the norm, even though it is inconvenient and requires extra stocks of PPE.
- Respiratory treatments: In perform aerosol-generating procedures (AGPs) in confirmed or suspected COVID-19 patients, practice enhanced droplet/contact precautions, including an N95 mask, eye protection, gown and gloves, or a powered air-purifying respirator. AGPs include intubation, extubation, bag mask ventilation, noninvasive ventilation (continuous positive airway pressure and bilevel positive airway pressure), airway suctioning, nebulizer therapies, bronchoscopy, chest tube insertion, thoracotomies, and pleural procedures.
- Transfers: Patients are best transferred directly to the OR, without stopping in the preoperative or post anesthesia care unit areas, to minimize exposure to other patients, staff, and other environments.
- “COVID-19 precautions” signs should be posted on all doors of the OR suite to inform staff of potential risks and minimize exposure.
- Negative pressure ventilation at more than 2.5 PA, at 12 or more air changes, can improve OR traffic movements and increase safety of patients and staff.
- In view of the possibility of false-negative COVID-19 testing (10%-30%), the American Society of Anesthesiologists recommends that all anesthesia professionals use PPE appropriate for AGPs for all patients during all diagnostic, therapeutic, and surgical procedures when working near the airway.
- If N95 masks are to be reused, ultraviolet germicidal irradiation, vaporous hydrogen peroxide, or moist heat by autoclaving may be used, if available.
- One staff member should adopt the role of being the donning-and-doffing observer because most nosocomial spread of COVID-19 occurs during this critical period.
- Limit entry/exit to a single OR entrance, keeping all OR doors closed as much as possible, and limiting staff entry/reentry to keep OR pressures and air exchanges regulated.
- Before AGPs are performed, OR personnel should ensure that no more than the minimal number of staff required to safely achieve the procedure are allowed to be in the room.
- Staff reliefs for breaks are essential to maintain focus and morale: these should be factored into the morning’s arrangements and organized to preserve PPE and minimize re-entries to the OR.
- Surgical approach and techniques may have to be reevaluated to optimize patient outcomes while minimizing exposure risk to providers. Use of laparoscopic or video-assisted thoracoscopic procedures may have to be deferred due to risk of aerosolization from CO2 insufflation systems^[25]^.
- A clear barrier screen is vital to limit aerosol transmission across the head of the table; limiting those who are allowed to touch equipment, syringes, medications, and crystalloid/blood products.
- Rapid sequence induction is preferable for airway management of a COVID-19+ or highly suspected patient. Induction can be performed according to usual airway management for non-COVID-19 patents.
- Limit the number of staff in the OR to the minimum needed to safely intubate (one anesthesiologist plus one or two assistants). Video-guided laryngoscopy may be chosen over direct visualization to decrease the risk of droplet transmission.
- Preoxygenate with 100% inspired oxygen and avoid bag-mask ventilation unless necessary. When resources permit it, patients are best recovered in a negative pressure isolation room (in the post-anesthesia care unit or ICU). Early recovery in the OR before transfer to a single patient room is favorable.
- After the patient has left the OR, the OR should be closed for an appropriate standoff period to achieve > 99.9% aerosol clearance. The amount of time that aerosols stay suspended in the air will depend on a number of factors, including the size of the room, the number of air changes per hour, how long the patient was in the room, whether the patient was coughing or sneezing, and whether or not an AGP was performed. General guidance on clearance rates under differing ventilation conditions is available from the CDC, United States of America.
- After the standoff period, the OR suite must be cleaned using routine procedures with approved hospital disinfectant on all surfaces, including drip stands and monitors.
- COVID-19 repeat testing is only required in postoperative patients when symptoms or signs of COVID-19 develop. Rapid polymerase chain reaction (or PCR) based COVID-19 testing is preferred when available.
- In the event of cardiac arrest or other medical emergency, all patients should continue to be treated as suspected or confirmed COVID-19 cases when performing cardiopulmonary resuscitation. This requires strict adherence to enhanced contact and droplet precautions. No patient interaction should occur before full PPE is donned. This paradigm shift for physicians accustomed to “jumping into lifesaving patient interactions with little regard to infectious risk” will be an uncomfortable transition. As best as it is possible within locations of LMICs, is helpful to follow the guidelines presented by many international societies like the International Liaison Committee on Resuscitation (or ILCOR), AHA, and Resuscitation Council UK. They offer regular interim updates and modified guidelines for resuscitation during COVID-19 pandemic^[26]^.
- Povidone iodine or popular mouthwashes and nasal rinse products are effective *in vitro* virucides against similar coronaviruses (SARS-CoV and Middle East respiratory syndrome-coronavirus) (although it has not been tested directly with COVID-19). They may have value when used as a preoperative mouth rinse, before surgery prior to transfer to the OR. Following induction, mouth or nasal painting with a sponge may also help to reduce risk of contamination to anesthesia personnel.
- Following reversal of heparinization, introduction of aspirin or fractionated heparin (as is used for hip surgery) may reduce the incidence of future thromboembolism.
- Nitric oxide may also help to reduce respiratory tract infection by inactivating viruses and inhibiting their replication in epithelial cells, but it is expensive and, in some locations, may be difficult to obtain^[27]^.

#### Transfer to Ward and Discharge

- Strictest adherence to bathroom cleanliness, minimizing sharing, is advised.
- Limiting those allowed to enter the hospital (one patient and one relative) will help reduce the traffic of people in vital care areas.
- If at risk patients are discharged early, the use of telecommunications for following up with them is recommended.
- Isolation with one supporting relative for 14 days following surgery may be the best way to limit complications and contamination by others in the household.

#### Neurological Issues (Before and After Cardiac Surgery)

- The risk of neurological damage as a result of cardiac surgery is a constant source of concern to cardiac surgeons in their endeavor to protect the cerebral and spinal circulation during the operation. It also has a potential for medico-legal intrusion. COVID-19 infection may complicate the clinical scenarios that are difficult to distinguish between COVID-19 and pre-existing cardiac issues now unmasked or altered despite a perfectly executed operation.
- While the pulmonary complications have received considerable attention, it is the neurological manifestations that are disabling, persistent, and common in patients infected with SARS-CoV-2. The entire neuro-axis can be involved resulting in a wide variety of manifestations. SARS-CoV-2 infection may be associated with encephalopathy and encephalomyelitis, ischemic stroke, intracerebral hemorrhage, anosmia, and neuromuscular diseases. The neurological status of the patient should therefore be carefully reviewed before and after surgery using identical evaluative clinical tools. Neurological screening checks need to be thorough and similar in all patients; routine interventional procedures and cardiac surgery may produce particle or air embolism despite protective measures. Recent studies with preoperative and postoperative MRI suggest that a large percentage of patients do suffer silent cerebral infarcts even though they appear to have had an uneventful procedure^[28]^.
- Preoperative checks: Perform clearly documented and detailed enquiry into any neurological symptoms, communication difficulties in verbal exchange, physical signs, and writing skills. Completion of consent forms and other routine documentation can help identify such deterioration. Repeat simple questions about date of birth, address information, and who is at home (which normally would have been previously obtained by reception staff) to determine limits on cognitive ability or impairment. This should include information about right or left-handedness, gait, and/or need for walking aids.
- Intraoperative check: A mutual check with anesthesia colleagues to ensure central line taps/spigots are luer-locked and can permit easy blood draw back before draping. Follow carefully planned myocardial protection, then ensure no air entrapment occurs that might migrate to the coronary arteries or cerebrally on completing the cardiac procedure.

#### Blood Products — Use of Donor Blood & Blood Type

Blood groups are increasingly recognized to influence susceptibility to certain viruses, among them SARS-CoV-1 and norovirus; individuals with A, B, and AB blood types may be at “increased risk for thrombosis and cardiovascular diseases”, which are important comorbidities among patients hospitalized with COVID-19^[29-32]^. Blood types A or AB in COVID-19 patients were associated with increased risk for needing mechanical ventilation, continuous renal replacement therapy, and prolonged ICU admission *vs.* patients with blood type O or B. Inflammatory cytokines levels did not, however, differ between groups in some studies. The virus which causes yellow fever (transmitted by fleas) is 40-50 nm in width and is transmissible through filtered human serum. The COVID-19 virus size ranges from 50-200 nm.

This raises the question as to the value of using blood products obtained by donation due to uncertain deleterious consequences. In view of the risk of viral transfer through standard filters, surgeons will have to give more attention to hemostasis, meticulous conservation of blood, and the principle that avoidance of blood and plasma use is desirable. Jehovah’s Witness patients have taught us to be more careful about hemostasis and that avoidance of donor blood can still usually lead to a safe outcome. Whether or not following this practice produces more certain and favorable outcomes in patients in which COVID-19 exposure is evident is currently uncertain. Leukoreduction of cellular blood products (particularly pre-storage leukoreduction done shortly after blood collection) is beneficial in prevention of multiple harmful effects of blood transfusion.

#### Legal Issues Arising from the Pandemic

This is a new pandemic. Adjustments to patient consent forms will be required for procedures and surgery so that healthcare personnel’s risk of liability is limited. CardioStart International has one that is legally approved of its international assistance to cardiac programs.

Healthcare workers who follow government guidelines in providing care in good faith during the public health emergency and in a reasonable time period may still not be adequately legally protected and the risk of ill-health among healthcare personnel is substantial.

#### The Near Future — A Revisionist Approach to Training Programs?

Few countries in resource-deprived settings have fully established training programs. The consequence of the COVID-19 pandemic for primary care providers in these settings is the loss of training and mentorship for members of these healthcare teams. The six leading modifiable CVD risk factors include high systolic blood pressure, diet, high low-density lipoprotein cholesterol, air pollution, high body mass index, and tobacco smoking. These are vitally important to identify, track, and counsel patients about, especially in communities living remotely from cities and the burden placed on healthcare workers throughout the community network will be much greater.

It is clear that established pediatric and/or adult heart programs will be imperiled: they require a full complement of suitably trained specialists (*i.e.*, cardiac surgeons, cardiologists, intensivists and anesthesiologists, nurses, perfusionists, and technicians in the OR and ICU) to retain a consistent and high standard of care. In some centers, colleagues will have to make do in a more restricted environment and will be reluctant to take on complex cases or teach a procedure. The potential for medico-legal risks will also likely rise which would lead to a reluctance to allow junior doctors to do cases under supervision.

## CONCLUSION

Every year, millions of people are dying and millions more are becoming disabled due to treatable cardiac surgical diseases. Surgical capacity is unevenly distributed around the world and disproportionally affects populations in LMICs. COVID-19 increases barriers for access to cardiac surgical care, and high-level action is urgently needed to not only expand cardiac services around the world but also to ensure health systems are strengthened so that cardiac patients can be effectively managed during and after the pandemic.

**Table:** 

Authors’ Roles & Responsibilities	
KP	Substantial contributions to the conception of the work; and the acquisition and analysis of data for the work; drafting the work; final approval of the version to be published
VN	Substantial contributions to the acquisition and analysis of data for the work; drafting the work; final approval of the version to be published
LB	Substantial contributions to the acquisition of data for the work; final approval of the version to be published
BF	Substantial contributions to the analysis and interpretation of data for the work; final approval of the version to be published
EAF	Substantial contributions to the acquisition and analysis of data for the work; final approval of the version to be published
NB	Substantial contributions to the acquisition of data for the work; final approval of the version to be published
RC	Substantial contributions to the design of the work; and the analysis of data for the work; final approval of the version to be published
DN	Substantial contributions to the acquisition and analysis of data for the work; final approval of the version to be published
AM	Substantial contributions to the conception and design of the work; or the analysis of data for the work; drafting the work; final approval of the version to be published
